# A Collaborative Approach to Mentored Peer Reviews Sponsored by the Council of Residency Directors in Emergency Medicine

**DOI:** 10.5811/westjem.61488

**Published:** 2023-12-06

**Authors:** Jeffrey N. Love, Chris Merritt, Jonathan S. Ilgen, Anne M. Messman, David P. Way, Douglas S. Ander, Wendy C. Coates

**Affiliations:** * Georgetown University School of Medicine, Department of Emergency Medicine, Washington, DC; † Brown University, Alpert Medical School, Department of Emergency Medicine, Providence, Rhode Island; ‡ University of Washington, Department of Emergency Medicine, Seattle, Washington; § Wayne State University, Department of Emergency Medicine, Detroit, Michigan; ∥ Ohio State University College of Medicine, Department of Emergency Medicine, Columbus, Ohio; ¶ Emory University, Department of Emergency Medicine, Atlanta, Georgia; # University of California: Los Angeles, David Geffen School of Medicine, Department of Emergency Medicine, Los Angeles, California

## Abstract

**Introduction:**

Historically, there have been no systematic programs for teaching peer review, leaving trainees to learn by trial and error. Recently, a number of publications have advocated for programs where experienced reviewers mentor trainees to more efficiently acquire this knowledge.

**Objective:**

Our goal was to develop an introductory learning experience that intentionally fosters peer-review skills.

**Methods:**

The Council of Residency Directors in Emergency Medicine (CORD) offered education fellowship directors the opportunity to mentor their fellows by reviewing submitted manuscript(s) supplemented by educational material provided by their journal. Reviews were collaboratively created. The decision letter that was sent to manuscript authors was also sent to the mentees; it included all reviewers’ and editor’s comments, as feedback. In 2022, fellows received a post-experience survey regarding prior experiences and their perspectives of the mentored peer-review experience.

**Results:**

From 2020–2022, participation grew from 14 to 30 education fellowships, providing 76 manuscript peer reviews. The 2022 survey-response rate of 87% (20/23) revealed that fellows were inexperienced in education scholarship prior to participation: 30% had authored an education paper, and 10% had performed peer review of an education manuscript. Overall, participants were enthusiastic about the program and anxious to participate the following year. In addition, participants identified a number of benefits of the mentored experience including improved understanding of the scholarship process; informing fellows’ scholarly pursuits; improved conceptualization of concepts learned elsewhere in training; and learning through exposure to scholarship.

**Conclusion:**

This program’s early findings suggest that collaboration between academic societies and interested graduate medical education faculty has the potential to formalize the process of learning peer review, benefitting all involved stakeholders.

## INTRODUCTION

The process of peer review has a longstanding history of providing both validity and credibility to published research.^1^ ^–^ ^3^ Traditionally, peer reviewers achieved competence through trial and error with some receiving unstructured mentorship from experienced reviewers.^2^ Although many have advocated for more rigorous and replicable processes for peer-review training, there remains a paucity of programs intentionally designed to achieve this goal.^2^ ^,^ ^4^ ^,^ ^5^

Over the last two decades, sporadic opportunities such as peer-review workshops, learning modules, and publications have been developed, yet these offerings have limited reach and variable content.^2^ ^,^ ^5^ ^,^ ^6^ More recently, a few authors have shared their experiences, advocating for mentored peer reviews (MPR) based on one-to-one interactions with more experienced reviewers^7^ ^–^ ^9^ and group peer reviews (GPR)^3^ ^,^ ^7^ ^,^ ^10^ ^,^ ^11^ that incorporate a mix of reviewer experiences. These approaches provide opportunities to learn peer review from experienced role models and to practice and refine skills alongside peers. Some programs have begun to make progress in formalizing the process of MPRs. A GPR program involving blogs in academic emergency medicine (EM) reported increased confidence among participants who also felt the process was friendly, easy, efficient, and transparent.^8^ The *Journal of the American College of Cardiology* similarly described a program in which fellows in a heart failure fellowship were nominated by an associate editor to learn the peer-review process through mentorship and group-based discussions.^7^

Although several editors in health professions education have expressed an interest in MPRs,^3^ ^,^ ^10^ ^,^ ^11^ we are not aware of any formal, larger-scale educational opportunities to train novice reviewers.

## OBJECTIVES

Cameron et al encouraged academic societies to sponsor professional development efforts related to education scholarship, including MPRs, which have the potential to “foster a pipeline of education scholars that reap benefits for an entire specialty.”^12^ In 2020 the Council of Residency Directors of Emergency Medicine (CORD) learned through a posting on the CORD listserv of a need among EM education fellowships for a learning opportunity related to peer review. A follow-up query on the CORD listserv yielded 14 education fellowships that were interested in having their fellows gain experience in this scholarly activity. As a result, CORD set about instituting learning communities around peer review, fostering MPR through the annual *Western Journal of Emergency Medicine Special Issue in Educational Research and Practice* (Special Issue). Consistent with CORD’s mission to “lead the advancement of emergency medicine education,”^13^ the objective of this opportunity was to develop an introductory, peer-review learning experience that would more intentionally foster peer-review skills. The data gathered as part of an observational study was used to provide a better understanding of the program’s growth and potential value to the participants and journal.

## CURRICULAR DESIGN

Fellowships in health professions education are becoming increasingly common as a means to provide junior faculty members with focused experiences in medical education practice and scholarship.^14^ ^–^ ^15^ Education fellowships within EM can be either one or two years in duration, the latter tending to have a more scholarly focus.^16^ Working closely with fellowship directors and other mentors, these programs offer an entrée into the community of practice of educators and education scholars through legitimate participation in teaching and education scholarship.^17^

Decision-making regarding curriculum development, program standards, and survey content were based on developing a consensus through an iterative process involving participating authors/editors. Mentored peer reviews were first offered to interested fellowship programs during the pilot phase in 2020. Fellowship directors received these offers as part of the normal rotation of reviewers, regardless of submission type or manuscript topic. Because education fellowships are not accredited by the Accreditation Council for Graduate Medical Education, they vary in structure and faculty support. Consequently, each fellowship director and mentee determined their own process of MPRs and negotiated how many reviews were appropriate each year.

At the end of every calendar year, editors solicited feedback from fellowship directors and fellows regarding how the program could be improved. This feedback informed editors’ efforts to structure an enhanced program based on guiding principles of successful professional development initiatives including the following: 1) a basis in experiential learning; 2) the provision of feedback; 3) effective peer and colleague relationships; 4) well designed interventions following principles of teaching and learning; and 5) a diversity of educational methods within single interventions.^18^ At the end of the 2022 submission cycle, a survey was initiated that included questions about participants’ background and prior experience (Supplemental File 1).

As an experiential learning opportunity, the four components of Kolb’s learning cycle were incorporated to maximize learning:^19^

• Concrete Learning: As a pre-interventional activity, we provided each mentee and their fellowship director with the following resources: three articles from varying perspectives on the principles of performing high quality peer review^20^ ^–^ ^22^; the scoring rubric editors used to assess reviews (Supplemental File 2); and a blinded copy from the Special Issue archives previously recognized as a quality review.
• Experimentation: Mentorship is recognized as an important influence on learning, research, productivity, personal development, and satisfaction.^23^ ^,^ ^24^ In our mentored peer-review process, novice peer reviewers play an authentic role in education scholarship under the guidance of a mentor, further incorporating them into a community of practice around shared values^25^ while promoting their professional identify formation.^17^ ^,^ ^25^ ^–^ ^28^
• Reflection: There were multiple opportunities for novice reviewers to reflect on their review experiences. This began with their discussions with mentors regarding the merits and potential areas of improvement for each article and continued in their individual and collective efforts to convey this feedback in written form as they constructed their reviews. When editors rendered a disposition for each manuscript, reviewers were copied on the decision letter sent to the authors. This letter summarized the factors important in the editor’s decision and included all reviewers’ comments. This approach has been advocated to promote reflection through other reviewers’ insights, and how the reviews were used collectively by the decision editor to render a decision.^20^
• Abstract Conceptualization: Professional development initiatives are most effective if they are integrated into a curriculum that allows for abstract conceptualization through reinforced learning and the opportunity to connect what was learned to related concepts.^11^ ^.^ ^25^ ^,^ ^29^ Integration of the CORD MPR program into the fellowships’ curricula enabled synergistic learning between the experiential learning afforded by the peer-review experience and underlying educational theory, best practices and research methodology, which are typical learning outcomes in education fellowships.

In the initial letter confirming acceptance of the review sent to mentor and mentee, we explicitly stated that the peer review was to be a mentored process with the final version representing a consensus perspective of those involved in the MPR. A single rating was provided for each MPR using our holistic editorial scoring rubric for reviews. Upon completing the initial peer review, participants were encouraged to perform additional mentored peer-reviews over the course of their fellowship training.

Our study of the Special Issue’s MPR program was determined to be exempt by the George Washington School of Medicine Institutional Review Board.

## IMPACE/EFFECTIVENESS

Over the three years of this intervention (2020–2022), participation grew from 14 to 30 education fellowships providing 58 fellows with the opportunity to participate in an MPR. The growth of the program over the first three years reflects a need among fellowship directors to provide a formalized educational experience in peer-review.

Twenty of the 23 (87.0%) participating fellows responded to the survey at the conclusion of the 2022 cycle regarding their background and prior experience (Table 1). Based on this survey, we learned that participants were novices with little experience in publishing or peer review. The fact that 80% of fellows were participating in a fellowship leading to a master’s degree reflects a cohort committed to a career in education scholarship. The value of this experience to participants is supported by the fact that 100% of survey respondent affirmed that the inclusion of the decision letter was helpful to their education and remained interested in serving as a peer reviewer for the following year’s Special Issue. We are in the process of contacting fellowship directors of graduating fellows to determine whether the mentors feel that their mentees are ready for independent peer review or whether they might benefit from additional mentored review experiences in the coming year.

**Table 1.** Background data of participating fellows who responded to the 2022 *Western Journal of Emergency Medicine Special Issue* call for participation in a mentored peer-review program.

Post-survey fellow questions	Yes/No #/Percentage
Have you authored a peer-reviewed publication related to education scholarship?	No 14/20 (70%)
Do you have prior experience performing peer reviews for publication?	No^a^ 18/20 (90%)
Did you participate in a formal education scholar track in your residency?	No 14/20 (70%)
Have you participated in a postgraduate education scholarship program (other than your current fellowship)?	No^b^ 17/20 (85%)
Will you be earning a master’s degree with your fellowship?	Yes 16/20 (80%)

a The two fellows having prior experience with peer reviews were from previous participation with this program.

b The three fellows with prior experience in postgraduate education scholarship programs were all participants in the American College of Emergency Physicians Teaching Fellowship.

Twenty of 23 participants also responded to the open-end question requesting suggested feedback for improving the program (Table 2). Although the suggestions made had little to do with improving the program, the responses provided were positive and enthusiastic regarding the value of the program. A number of these comments reflected potential benefits of the mentored peer-review experience including the following: learning content through critiquing articles with emerging questions and background information; better understanding of the peer-review process; improving the quality of the fellows’ future scholarly submissions; and serving the role of abstract conceptualization in fellow learning.

**Table 2.** Emergency Medicine fellows’ responses on the 2022 post-program survey to the open-ended question, “Please provide any feedback that would improve the value of the mentored peer-review program as a learning experience”.

I found the attached articles very helpful in supplementing my knowledge and aiding me in my review. I have referred to them when doing review for another journal since this experience.
This experience was extremely helpful in better understanding the role of peer-review in decision making regarding publication as well as likely improving the quality of my future scholarly submissions.
I thought the mentored peer review program was excellent. When the program started multiple materials including peer review guidelines and information on what to focus on during the review process were provided. There was easy communication to editors for clarification of questions. It gave me several opportunities to review current educational research articles, spend time to critically think about both the research itself, ensuring that research met the criteria to be high quality projects, that educational theory was used, and to identify whether the manuscripts were submitted within the guidelines required for the journal. I also appreciated being able to review a qualitative analysis manuscript. The only area for improvements I think may be useful is to provide some more opportunities to learn from the editors’ perspective. For example, what do you prioritize in making a final decision on a manuscript? Are there any resources apart from those initially provided that are commonly referenced for specific educational themes or for certain kinds of studies? Just some ideas to get further insight into the thought process that goes into making a final decision on a submission. Thank you!
I anticipate working next year at a resident site in XXXX. They do not have a Med Ed Fellowship, but I would be happy to continue reviewing while there.
Really positive experience overall – really like this as an introduction to peer review!
This was an excellent formative activity. Thank you for this opportunity!
This was a great experience, thank you for the opportunity. I would be happy to review in either a mentored or independent fashion in the future.
The experience was valuable in getting experience performing peer review. I would love the opportunity to participate again!
Overall, a great experience and helped me to see the publication process from the inside-and think it will help me strengthen my own future publications.
Thank you for the chance to review.
I thought the process was very smooth! I found the attached documents on how to review a manuscript and tips very helpful especially as a first-time reviewer.

Over the period of this study, the CORD MPR program provided 76 external peer reviews, 79% as mentor-mentee dyads and 21% as GPRs. The number of peer reviews provided by participating fellows from 2020–2022 ranged from 1–6 with an average of 1.6 reviews per fellow. A consensus discussion of the editors in each of the past three years concluded that the overall quality of the mentored peer reviews was very good to excellent, suggesting the value of this experience to the journal. This conclusion was substantiated by the fact that 50% (10/20) of those reviews recognized in 2020 as outstanding (editorial score of 5 on a 5-point scale used by the journal) were authored by 10 of the 14 (71.4%) fellowships participating in the mentored peer-review program.

The variability across programs in how the mentoring process was carried out limits what can be concluded regarding the appropriateness of the various approaches used.

### Lessons Learned

Early in the 2022 submission cycle, the potential of this experience to serve as an introduction to the education scholarship community of practice as well as contribute the professional identify formation of fellows was apparent to the editors. With this in mind, in 2022 the fellow was made the point person for questions to the editor that had not been answered by advanced reading material or by the fellowship director as well as being responsible for submitting the review. This appeared to empower the fellows as they initiated appropriate questions about the peer-review process, expectations and outcomes to a greater degree than had previously been experienced with traditional reviewers.

Although overall the number of fellowships taking part in the program increased steadily over time, the editors noted that participation of interested fellowship programs appeared unpredictable. Through follow-up with the programs, we learned that this issue was often related to the timing of review offers, which did not always align with the fellows’ training schedules. At the beginning of the 2022 cycle, we asked each fellowship director to provide optimal time periods to send requests. This appeared to significantly improve the number of programs that participated.

From an administrative standpoint, this program required a significant time commitment from the journal’s editorial staff to track fellowship programs’ availability, forward educational materials, and manage follow-up. Although this commitment may be considered limiting, the editors viewed it as an investment in the future of our community, the journal, and as a service to the academic community at large to provide an enhanced pool of trained and qualified peer reviewers.

## CONCLUSION

Early outcomes of the CORD mentored peer review program are encouraging, addressing a previously unmet need for sustainable reviewer training that could benefit academic journals and reviewers alike. Our cohort of novice reviewers reported multiple learning benefits across this experience, from a more scaffolded approach to peer reviewing as well as opportunities to reflect on their own scholarship. This suggests a climate that supports ongoing participation, more rigorous independent review, and rigorous education research.

Several studies of the program are currently underway to evaluate the value of the CORD MPR program to major stakeholders including the journal, editors, and authors. Although early outcomes of this work suggest several purported benefits of MPRs, a richer understanding of the value of this experience to the participants is needed, and qualitative explorations with mentees are underway.

Future studies are also needed to determine the long-term benefits of the program. Additional research will determine the degree to which the CORD MPR program may generalize to other journals, academic societies and graduate medical education in general. Although having an existing journal partnership facilitated our ability to shape and study this experience, recent interest in MPRs suggests the potential to develop such partnerships for others. This program’s early findings suggest that collaboration between academic societies and interested graduate medical education faculty have the potential to formalize the process of learning peer review to the benefit of all involved stakeholders.

## Supplementary Information




