# Supplementary material for: A Collaborative Approach to Mentored Peer Reviews Sponsored by the Council of Residency Directors in Emergency Medicine
Source: West J Emerg Med. 2023 Dec 6;25(1):111–6. doi: 10.5811/westjem.61488 (PMC10777179; doi:10.5811/westjem.61488)
Supplement: Supplementary file 1 [file wjem-25-111-s001.docx]

Supplemental File 1

Holistic Editorial Scoring Rubric

Gold Standard Reviews are those that provide:

1. Insightful review (detailed and global) that reflects upon how the work under consideration may be of value to the readership and informs the current literature.
2. Appropriateness of the study method(s) employed and a reflection of the relevant tenets of education scholarship.
3. Feedback that provides mentorship to authors on how to improve their manuscript and their own skill set.

**5-Exceptional:** A model review that reflects each of the tenets of the Gold Standard and could stand alone as a summary to the authors. Recommendations to the authors are appropriate, actionable and supportive with a basis in educational scholarship. The review provides an in-depth perspective which may include relevant citations, resources or specific suggestions for improving the manuscript and/or professional growth. An additional contributing factor includes instances where the reviewer makes an important observation or recommendation not previously considered by the editors.

**4-Very Good:** An excellent review that reflects the time, effort and expertise necessary to contribute substantially to the final formal decision but falls short in one or more of the 3 key areas that define the “Gold Standard”. For example, an excellent overall review that (1) misses 1-2 substantive points, (2) provides only cursory mention of educational scholarship concepts or (3) falls short of providing mentoring support when critiquing the authors work.

**3-Good:** The review meets the standard of an acceptable review. The analysis adds to the broader perspective in a measured way but is not as complete, organized, documented or is lacking adequate explanations for the authors. As a result, additional reviews are required to provide more extensive/actionable feedback to the authors.

**2-Below Average:** Though there may be some insights included the review provides a superficial evaluation of the submission. This may include lack of reasoning for the decisions rendered, comments are not actionable or there may be a general lack of critique for improvement. In essence, insights provided may reinforce other reviewers’ comments but are not substantive enough to shape editorial decision-making pertaining to the manuscript. The majority of components of a “Gold Standard review” are missing.

**1-Unacceptable:** The review is sparse and may provide 1-2 insights but either (1) provides a decision without explanation (accept/reject, like/dislike, good/bad) (2) provide praise without critique (no substantive feedback for how to improve the manuscript) and concludes “accept as is” when revisions are needed or rejects with minimal justification, (3) lacks meaningful insights or (4) conclusions are based on faulty reasoning (i.e. disagrees with opinions of other reviewers and the editor). In short, the review provides little if any substantive critique that contributes to consensus decision making.
